# ECG Heartbeat Classification Using Machine Learning and Metaheuristic Optimization for Smart Healthcare Systems

**DOI:** 10.3390/bioengineering10040429

**Published:** 2023-03-28

**Authors:** Mahmoud Hassaballah, Yaser M. Wazery, Ibrahim E. Ibrahim, Aly Farag

**Affiliations:** 1 Department of Computer Science, College of Computer Engineering and Sciences, Prince Sattam Bin Abdulaziz University, AlKharj 16278, Saudi Arabia; 2 Faculty of Computers and Information, Minia University, Minia 61519, Egypt; 3 Faculty of Computers and Information, Luxor University, Luxor 85951, Egypt; 4 Department of Electrical and Computer Engineering, University of Louisville, Louisville, KY 40292, USA

**Keywords:** smart healthcare, patient health monitoring, ECG classification, IoT sensors, metaheuristic algorithms, supervised learning

## Abstract

Early diagnosis and classification of arrhythmia from an electrocardiogram (ECG) plays a significant role in smart healthcare systems for the health monitoring of individuals with cardiovascular diseases. Unfortunately, the nonlinearity and low amplitude of ECG recordings make the classification process difficult. Thus, the performance of most traditional machine learning (ML) classifiers is questionable, as the interrelationship between the learning parameters is not well modeled, especially for data features with high dimensions. To address the limitations of ML classifiers, this paper introduces an automatic arrhythmia classification approach based on the integration of a recent metaheuristic optimization (MHO) algorithm and ML classifiers. The role of the MHO is to optimize the search parameters of the classifiers. The approach consists of three steps: the preprocessing of the ECG signal, the extraction of the features, and the classification. The learning parameters of four supervised ML classifiers were utilized for the classification task; support vector machine (SVM), k-nearest neighbors (kNNs), gradient boosting decision tree (GBDT), and random forest (RF) were optimized using the MHO algorithm. To validate the advantage of the proposed approach, several experiments were conducted on three common databases, including the Massachusetts Institute of Technology (MIT-BIH), the European Society of Cardiology ST-T (EDB), and the St. Petersburg Institute of Cardiological Techniques 12-lead Arrhythmia (INCART). The obtained results showed that the performance of all the tested classifiers were significantly improved after integrating the MHO algorithm, with the average ECG arrhythmia classification accuracy reaching 99.92% and a sensitivity of 99.81%, outperforming the state-of the-art methods.

## 1. Introduction

The recent developments in biomedical sensors, the Internet of Medical Things (IoMT), and artificial intelligence (AI)-based techniques have increased interest in smart healthcare technologies [1,2]. Microelectronics, smart sensors, AI, 5G, and IoMT constitute the cornerstone of smart healthcare [3,4]. A smart healthcare system does not suffer fatigue; hence, it can process big data at a much higher speed than humans with greater accuracy [5]. With smart healthcare systems, the diagnosis and treatment of diseases have become more intelligent. For instance, smart patient monitoring empowers the observation of a patient outside the traditional clinical settings, which offers a lower cost through reducing visits to physician offices and hospitalizations [6].

The human body is known as a complex electromechanical system generating several types of biomedical signals, such as an electrocardiogram (ECG), which is a record of the dynamic changes of the human body that need to be monitored by smart healthcare systems. For instance, the EKG sensor measures cardiac electrical potential waveforms. It is used to create standard 3-lead electrocardiogram (EKG) tracings to record the electrical activity in the heart or to collect surface electromyography (sEMG) to study the contractions in the muscles of the arm, leg, or jaw. Simply, an ECG graphs heartbeats and rhythms. The classification of an ECG heartbeat plays a substantial role in smart healthcare systems [7,8], where the presence of multiple cardiovascular problems is generally indicated by an ECG. In the subsequent ECG waveform, diseases cause defects. However, early diagnosis via an ECG allows for the selection of suitable cardiac medication and is thus very important and helpful for reducing heart attacks [9]. The method of detecting and classifying arrhythmia is not an easy task and may be very difficult even for professionals because sometimes it is important to examine multiple pulses of ECG data, obtained, for example, during hours, or even days, by a Holter clock. Furthermore, there is a possibility for errors by humans during the ECG recording study due to fatigue. Building a fully automatic arrhythmia detection or classification system is difficult. The difficulty comes from the large amount of data and the diversities in the ECG signals due to the nonlinearity, complexity, and low amplitude of ECG recordings, as well as the nonclinical conditions, such as noise [10].

Despite all these difficulties, methods for ECG arrhythmia classification have been widely explored [11,12] but choosing the best technique for smart patient monitoring depends on the robustness and performance of these methods. Several convolutional neural network (CNN)-based approaches have been introduced for the task [13,14]. Bollepalli et al. [10] proposed a CNN-based heartbeat detector to learn fused features from multiple ECG signals. It achieved an accuracy of 99.92% on the MITBIH database using two ECG channels. In [15], a subject-adaptable ECG arrhythmia classification model was proposed and trained with unlabeled personal data. It achieved an average performance of 99.4% classification accuracy on the MIT-BIH database. In [16], an end to-end deep multiscale fusion CNN model of multiple convolution kernels with different receptive fields was proposed, achieving an F1 score of 82.8% and 84.1% on two datasets. Chen et al. [17] combined CNN with long short-term memory to classify six types of arrhythmia and achieved an average accuracy of 97.15% on the MIT-BIH database. A recent approach by Atal and Singh [18] proposed using the bat-rider optimization to optimally tune a deep CNN to achieve an accuracy of 93.19% with a sensitivity of 93.9% on the MIT-BIH database. Unfortunately, most CNN-based methods are effective only for small numbers of arrhythmia classes, are computationally intensive, and need a very large amount of training data [13]. This is a great challenge for using the CNN-based methods on real-time applications or wearable devices with limited hardware [19].

On the other hand, many research efforts have been devoted to ECG arrhythmia classification using ML classifiers, such as SVM, RF, kNN, linear discriminants, multilayered perceptron, and regression tree [20,21]. It is well known that the SVM classifier does not become trapped in the well-known local minima points, requires less training data, and is faster than CNN-based methods [22]. In [23], wavelet transform and ICA were used for the morphological features description of the segmented heartbeats. The features were fed into an SVM to classify an ECG into five classes. In [24], least square twin SVM and kNN classifiers based on features’ sparse representation were used for cardiac arrhythmia recognition. The experiments were carried out on the MIT-BIH database in category and personalized schemes. A method based on improved fuzzy C-means clustering and Mahalanobis distance was introduced in [25], while in [26], abstract features from abductive interpretation of the ECG signals were utilized in heartbeat classification. Borui et al. [27] proposed a deep learning model integrating a long short-term memory with SVM for ECG arrhythmia classification. Martis et al. [28] evaluated the performance of several ML classifiers and concluded that the kNN and higher-order statistics features achieved an average accuracy of 97.65% and sensitivity of 98.16% on the MIT-BIH database. In [29], the RF classifier was utilized with CNN and PQRST features for arrhythmia classification from imbalanced ECG data. The major drawback of ML classifiers (e.g., SVM) is their deficiency in interpreting the impact of ECG data features on different arrhythmia patterns for extracting the optimal features. Further, the performance of most ML classifiers is questionable because the interrelationship between the learning parameters is not well modeled, especially for data features with high dimensions.

Despite the large amount of previous studies in the field, ECG arrhythmia classification has not been completely solved and remains a challenging problem. Consequently, there is room for improvement in several aspects, including classification, feature extraction, preprocessing, and ECG data segmentation. Most ML classifiers have some limitations; for example, SVM does not perform well with noisy data, while random forest (RF) suffers from interpretability issues and fails to determine the significance of variables. In addition, these ML classifiers have many parameters, and tuning such parameters has a crucial influence on the efficiency of the classification. Motivated by the advantages of the ML classifiers compared to the CNN-based methods, although they face a major challenge with a low classification accuracy, in this work, we focus on enhancing the classification accuracy of the ML classifiers. To this end and to develop an efficient classifier model, we propose to optimize the learning parameters of these classifiers using a naturally inspired metaheuristic algorithm called the marine predators optimization algorithm (MPA). The parameters of the classifier are gradually optimized using the MPA algorithm, which introduces an optimal classifier model that can classify the ECG features efficiently. Four different machine learning classifiers are considered, namely SVM, GBDT, RF, and kNN. The performance of these classifiers without learning parameter optimization and with optimization (i.e., MPA-SVM, MPA-GBDT, MPA-RF, and MPA-kNN) are compared. The experiments are validated on the three common benchmarking databases: the MIT-BIH, EDB, and INCART.

The remainder of this paper is organized as follows. Section 2 presents the methodology proposed to classify the ECG arrhythmia based on the optimization of the parameters of the ML classifiers. The experimental results and analysis as well as a comparison with the state of the art are presented in Section 3. Finally, the paper is concluded in Section 4.

## 2. Methodology

**Figure 1:**
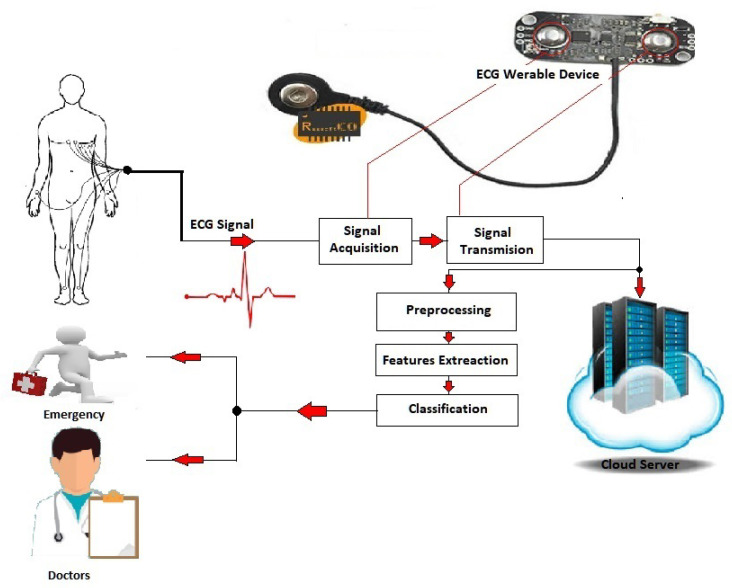
Overview of an early diagnosis and classification of ECG arrhythmia healthcare system.

A complete smart healthcare system consists of several parts, such as sensors for heartbeat recordings, dry electrodes sensing of heartbeats, interpretation of the heartbeat signals, a personalized system for heartbeat monitoring, and incorporation of the heartbeat monitoring system into healthcare. The overview of an early diagnosis and classification of ECG arrhythmia healthcare system is illustrated in Figure 1. It consists of three main steps: data preprocessing, feature extraction, and classification. Detection or classification is the vital step in the system; thus, the contribution of this work is mainly in the classification step as explained in the following.

### 2.1. Data Preprocessing and Feature Extraction

Denoising and reliable segmentation increase the efficiency of the classifiers [30], where the frequency of an ECG is between 0.5 Hz and 50 Hz [31]. To eliminate disturbances from the digital ECG signal, an FIR band-pass filter [32] designed with cutoff frequencies was utilized for this task. For the segmentation task of the ECG, the R-peaks annotations described by the MIT-BIH, EDB, and INCART datasets were considered as an indication of the beats segmentation, and for every beat, we centered a patch of size 200 ms around its R-peak within 75 ms before the R-peak and 110 ms after the R-peak.

After the segmentation phase, the features were extracted around the regions of the segmented ECG signal. In this work, different techniques were used for the feature extraction phase, including the 1D-local binary pattern (LBP) [33], higher-order statistics (HOS) [34], discrete wavelet transform (DWT), the Hermite basis function (HBF) [35], the central moment (CM), and the R-R intervals. Table 1 outlines the number of extracted features by each descriptor from the MIT-BIH, EDB, and INCART databases.

### 2.2. Classification

The supervised machine learning classifiers considered for detecting rhythm diseases with a number of parameters optimized using the proposed artificial intelligence metaheuristic optimization (MHO) algorithm were the SVM, random forest (RF), gradient boosting decision trees (GBDT), and K-nearest neighbor (kNN).

#### 2.2.1. Support Vector Machine

The SVM offers strong insight for practical applications and contributes to high efficiency [36]. It works by transforming input data from the basic domain P into a new higher dimension feature space; thereafter, it searches in this space for the optimal hyperplane. It aims to split the training data into groups in order to find a maximum marginal hyperplane. Mathematically, an instance $x_{i}$ is connected with a label $y_{i}\in \left\{+1,-1\right\}$. The hyperplane divides the multidimensional space into negative and positive instances induced by the kernel function with the maximum margin and the minimum classification [37]. Suppose z=φ(x) is a feature space vector, and $w_{i}$ maps φ from P to the feature space Z; then, the hyperplane is

(1) w·z+b=0,

defined by the pair (w,b), which is obtained by separating the point $X_{i}$, such that

(2) $$f\left(x_{i}\right)=\mathrm{sign}\left(w\cdot z_{i}+b\right)=\left\{\begin{array}{cc} 1, & \;\mathrm{if}\;x_{i}=1\\ -1, & \;\mathrm{if}\;x_{i}=-1 \end{array},\right.$$

where w∈Z and b∈P. *S* is linearly separable, if there is (w,b) with

(3) $$\left\{\begin{array}{cc} \left(w\cdot z_{i}+b\right)\geq 1, & \;\mathrm{if}\;x_{i}=1\\ \left(w\cdot z_{i}+b\right)\leq -1, & \;\mathrm{if}\;x_{i}=-1, \end{array},where\;\;i=1,\ldots ,l\right.$$

is applicable for each element on *S*. If *S* is not linearly separable, the SVM formulation must allow for classification violations. The ideal with a hyperplane is a solution to

(4) $$\begin{array}{c} \mathrm{minimize}\frac{1}{2}w\cdot w+C\sum_{i=1}^{l}\xi_{i},\\ \;\mathrm{such}\;\mathrm{that}\;y_{i}\left(w\cdot z_{i}+b\right)\geq 1-\xi_{i},,\;\mathrm{where}\;\xi_{i}\geq 0,\;i=1,\ldots ,l. \end{array}$$

#### 2.2.2. Gradient Boosting Decision Tree

In decision trees, every internal node is labeled with a distinctive input. The arcs derived from the node marked with a certain feature are labeled with each of the possible feature values. Every tree leaf is classified as having a class or distribution of probability over classes. The basic concept of the decision tree for gradient boosting is to combine a series of weak base classifiers into one strong classifier. Unlike traditional methods of boosting samples that weigh positive and negative, the GBDT uses global algorithm convergence by following the negative gradient direction. The weak learner estimates the error at every splitting node based on a test function $\kappa :R^{n}\to R$ considering a threshold τ for the returns $\eta^{l}$ and $\eta^{r}$. The optimal split is achieved by identifying the triplet $\left(\tau ,\eta^{l},\eta^{r}\right)$ that minimizes the error after the split, where

(5) $$\epsilon \left(\tau \right)=\sum_{i:\kappa \left(x_{i}\right)<\tau }w_{i}^{j}{\left(r_{i}^{j}-\eta^{l}\right)}^{2}+\sum_{i:\kappa \left(x_{i}\right)\geq \tau }w_{i}^{j}{\left(r_{i}^{j}-\eta^{r}\right)}^{2}.$$

The weight $w_{i}^{j}$ and response $r_{i}^{j}$ of $x_{i}$ at an iteration *j* are

(6) $$\begin{array}{c} w_{i}^{j}=\exp\left(-y_{i}f_{j-1}\left(x_{i}\right)\right),\\ r_{i}^{j}=g\left(x_{i}\right)/w_{i}^{j}=-y_{i}\exp\left(-y_{i}f_{j-1}\left(x_{i}\right)\right)/w_{i}^{j}=-y_{i}. \end{array}$$

#### 2.2.3. Random Forests

RF is close to the Bayesian method and is used to recognize an ensemble with a combination of hierarchical tree structure predictors [38]. The basic concept behind the RF is that a set of learning tree models may perform well compared to single decision trees if they make uncorrelated mistakes. In this context, we develop several trees instead of a single tree, where each tree is constructed upon values of random vectors sampled independently following the whole forest distribution. Consequently, the RF is an ensemble classifier consisting of many random decision trees. A single classification output of these decision trees is taken, and the values are collected to produce the final result of the classifier [39]. The RF, once constructed, is very fast, as it requires little computation. It has clear interpretability, which provides a natural way to incorporate prior knowledge. Employing appropriate randomness produces precise regressors and classifiers. Moreover, some studies have shown that random input features result in a high classification performance [40].

#### 2.2.4. K-Nearest Neighbor

The kNN is one of the straightforward and simplest machine learning schemes based on supervised learning. It is a non-parametric technique, which means that the underlying data do not need any assumptions during data classification. It implies the similarity between the new class and available instances and puts the new class in the category that is most closely related to the available categories. Generally, the estimation that can be obtained with the kNN scheme is prone to local noise and not very satisfactory. The larger the value of *k*, the smoother the classification boundary, while a smaller *k* is more convoluted to the boundary. An advantage of the kNN is that there is no training required.

### 2.3. Marine Predator Algorithm (MPA)

The MPA is a naturally inspired metaheuristic algorithm that imitates the behavior of predators to catch their victim or prey, employing two techniques when targeting their prey (Brownian and Lévy) [41].

#### 2.3.1. Initialization

Similar to all the metaheuristic schemes, the algorithm begins with an initial solution uniformly distributed over the search space such that

(7) $$Y_{0}=Y_{L}+\;\mathrm{rand}\;\left(Y_{U}-Y_{L}\right),$$

where $Y_{U}$ and $Y_{L}$ are the minimum and maximum boundary limits of the search spaces, respectively.

#### 2.3.2. Elite and Prey Matrix Construction

It is employed to construct an n×d Elite matrix *E* with

(8) $$E=\left[\begin{array}{cccc} Y_{1,1}^{I} & Y_{1,2}^{I} & \ldots & Y_{1,d}^{I}\\ Y_{2,1}^{\prime } & Y_{2,2}^{I} & \ldots & Y_{2,d}^{\prime }\\ \vdots & \vdots & \vdots & \vdots \\ Y_{n,1}^{I} & Y_{n,2}^{I} & \ldots & Y_{n,d}^{I} \end{array}\right],$$

where *n* refers to the number of search space agents, and $\vec{y_{I}}$ symbolizes the superior predator vector iterated *n* times to create the matrix. The prey matrix with the same dimensions *d* as the Elite is

(9) $$\;\mathrm{Py}\;=\left[\begin{array}{cccc} Y_{1,1} & Y_{1,2} & \ldots & Y_{1,4}\\ Y_{2,1} & Y_{2,2} & \ldots & Y_{2,d}\\ Y_{3,1} & Y_{3,2} & \ldots & Y_{3,d}\\ \vdots & \vdots & \vdots & \vdots \\ \vdots & \vdots & \vdots & \vdots \\ Y_{n,1} & Y_{1,2} & \ldots & Y_{n,d} \end{array}\right].$$

The optimization process in the MPA is mainly based on these two metrics, where the initialization generates the starting prey, from which the optimal fit builds this elite matrix.

#### 2.3.3. Optimization Process

The most critical step in which the predators seek to find the optimal fit or solution is the optimization cycle. In the discovery phase, which is the starting point, the predators attempt to move faster before they detect the prey such that:

For $t<\frac{1}{3}*t_{\max}$

(10) $$\begin{array}{c} \;{\vec{stepsize}}_{j}={\vec{R}}_{L}⊗\left({\vec{E}}_{j}-{\vec{R}}_{L}⊗\vec{py_{j}}\right) \end{array}$$

(11) $$\begin{array}{c} {\vec{\;\mathrm{Py}\;}}_{j}={\vec{\;\mathrm{Py}\;}}_{j}+P\cdot R⊗stepsize_{j}, \end{array}$$

where the vector ${\vec{R}}_{L}$ consists of the random values computed by the Lévy distribution, which represents the Lévy movements. Meanwhile, the process of the multiplication of the ${\vec{R}}_{L}$ and elite symbolizes the movements of the predators in the Lévy scheme, while utilizing the phase size for the elite position mimics the movements of the predators updating the position of the prey.

In the middle stage, the algorithm divides the population into two portions to distinguish the difference between exploration and exploitation. In this stage with $\frac{1}{3}*t_{\max}<t<\frac{2}{3}*t_{\max}$, the half population is

(12) $$\vec{stepsize_{j}}={\vec{R}}_{L}⊗\left({\vec{E}}_{j}-{\vec{R}}_{L}⊗\vec{py_{j}}\right)$$

(13) $${\vec{py}}_{j}={\vec{py}}_{j}+P*\vec{R}⊗\vec{stepsize_{j}},$$

while in the second half, it is

(14) $$\vec{stepsize_{i}}={\vec{R}}_{B}⊗\left({\vec{R}}_{B}⊗{\vec{E}}_{j}-{\vec{P}}_{j}\right)$$

(15) $${\vec{py}}_{j}={\vec{E}}_{i_{j}}+P*CF⊗\vec{stepsize_{j}},$$

and

(16) $$CF={\left(1-\frac{t}{t_{\max}}\right)}^{\left(2\frac{t}{t_{\max}}\right)}.$$

The population is modified using Lévy flight with $t>\frac{2}{3}*\max$-iter

(17) $${\vec{stepsize}}_{j}={\vec{R}}_{L}⊗\left({\vec{R}}_{L}⊗{\vec{E}}_{j}-\vec{py_{j}}\right)$$

(18) $${\vec{py}}_{j}={\vec{E}}_{j}+P*CF⊗{\vec{stepsize}}_{j}.$$

The predators accurately remember the previous locations of successful foraging because of their good memory. Using memory saving, the MPA algorithm simulates this ability of remembering successful foraging places, which can increase the quality of the solutions with the increase in iterations. Solution fitness at the present iteration is matched with its counterpart in the previous one. The new one replaces the solution if it is more suitable. The steps of the MPA are summarized in Algorithm 1.

#### 2.3.4. Parameters Optimization

Using the MPA algorithm, four optimized versions of the ML classifiers were introduced for ECG signal classification, namely the MPA-SVM, MPA-GBDT, MPA-RF, and MPA-KNN. The fitness function acts according to each classifier and its parameters. Tuning parameters has a crucial influence on the efficiency of the classification. Thus, a diverse set of parameters for each classifier was considered to optimize the classification stage. To fine-tune the best value for the parameters, the holdout strategy was considered with 80% for the training set, and the remaining 20% was used to test the performance. The list of parameters considered in the experiments for each classifier is provided in Table 2.
**Algorithm 1** Pseudo-code of MPA algorithm.  1: Initialization step, P, TP, TF, ${Py}_{i}$.  2: **while** t $<t_{max}$ **do**  3:     Compute the fitness value of each ${\vec{py}}_{i},f\left({\vec{py}}_{i}\right)$  4:     Construct *E*  5:     Implement the memory saving  6:     Update CF using Equation (16)  7:     **for** each $py_{i}$ **do**  8:     **if** $\left(t<\frac{1}{3}*t_{\max}\right)$ **then**  9:        Reposition the current ${\vec{py}}_{i}$ based on Equation (11)10:     **else**11:        **if**  $\left(\frac{1}{3}*t_{\max}<t<\frac{2}{3}*t_{\max}\right)$ **then**12:         **if** $\left(i<\frac{1}{2}*n\right)$ **then**13:         Reposition the current ${\vec{py}}_{l}$ using Equation (13)14:         **else**15:         Reposition the current ${\vec{py}}_{i}$ using Equation (15)16:         **end if**17:        **else**18:         Reposition the current ${\vec{py}}_{i}$ using Equation (18)19:        **end if**20:     **end if**21:    **end for**22:    Compute the fitness value of each ${\vec{py}}_{i},f\left({\vec{py}}_{i}\right)$23:    Update TopPradatorPos, and TopPredatorFit.24:    Apply the memory saving25:    Apply the FADS for ∀ ${\mathrm{py}}_{i}$26:    t++27: **end while**

## 3. Experiments and Results

### 3.1. Database Descriptions

The main characteristics of the three ECG databases used in the evaluation process are summarized in Table 3.

#### 3.1.1. The MIT-BIH Arrhythmia Dataset (MIT-BIH)

This is a public dataset showing the regular investigation content of cardiac rhythm detection collected from 47 patients. It consists of 48 records, where each one is 30 min in duration, with a 360 Hz sample rate. Moreover, the records have two signals: the first is a bipolar limb lead named the modified lead (MLII), and the second one is related to unipolar chest leads called V leads (V1, V2, V3, V4, V5, and V6). The MLII type is shareable though all records because it provides an ideal view for the significant waves (e.g., Q-waves, P-waves, R-Waves, T-waves, and S-waves) [42].

#### 3.1.2. The European ST-T Dataset (EDB)

The EDB was planned to be used to evaluate the performance of ST and T-wave architectures. It is a collection of 90 annotated samples of patients’ ECG records taken from 79 subjects. Each record has a two hour duration with two signals recorded at 250 sps [43].

#### 3.1.3. St. Petersburg INCART Dataset (INCART)

This is a 12-lead arrhythmia dataset containing 75 annotated recordings taken from 32 Holter records, each 30 min long. The INCART consists of 12 regular leads, and each lead is sampled at 257 Hz. The main records were acquired from patients undergoing coronary heart disease examinations [43].

### 3.2. Evaluation Criteria

As a strategy of classification, the holdout strategy was used to evaluate the performance of the optimized ML classifiers against five standard criteria including accuracy (Acc), precision (Pr), specificity (Sp), sensitivity (Sn), and the F1-score (F1). The performance criteria typically rely on different major metrics (positive/negative/true/false) of a binary classification test as follows:

(19) $$Acc=\frac{Tp+Tn}{Tp+Fn+Fp+Tn}$$

(20) $$Sn=\frac{Tp}{Tp+Fn}$$

(21) $$Sp=\frac{Tn}{Fp+Tn}$$

(22) $$Pr=\frac{TP}{TP+FP}$$

(23) $$F1=\frac{TP}{TP+FP}$$

### 3.3. Performance Evaluation

#### 3.3.1. Evaluation of the MPA-SVM

For the MPA-SVM classifier, two parameters, *c* and gamma, which had important effects on the classification process, were optimized. According to Table 4, all measures were higher than 98%. Looking at the class level, it is clear that the ACC and Sn were high on three datasets, where the ACC >99.14%, and Se >98.11%. The accuracy of the classification process by all models was remarkably enhanced and nearly balanced for all classes. The best reported accuracy was obtained for the class F with 99.93%, and the lowest performance was obtained for the class N with an accuracy of 99.86. Regarding the misclassification, only ≤0.49% of the S class and ≤0.82% of the VEBs class were not classified accurately. The results in the case of the S and VEBs classes were very promising, signifying improvements over counterpart studies. These two classes are important cases for the AAMI, which recommends that evaluation measures should focus on the classification of the S and VEBs classes. The performance of the MPA-SVM classifier was sufficient for these two classes on the three databases.

#### 3.3.2. Evaluation of the MPA-GBDT

The MPA-GBDT was introduced to optimize three parameters, the max depth, gamma, and learning rate. Table 5 lists the results of the optimized parameters for the MPA-GBDT model for the AMMI classes (N, S, VEBs, and F). In addition to the high accuracy of classification (≥99.45), the classification performance (sensitivity of ≥98.49% and positive predictivity of ≥98.81%) for the S and VEBs classes were very high, where the positive predictivity reached 100% for the classes of the MIT-BIH and EDB databases.

#### 3.3.3. Evaluation of the MPA-RF

The proposed MPA-RF optimized the same parameters as for the MPA-GBDT. Table 6 reports the obtained results on the same databases with the same validation scheme, and the MPA-RF provided the highest accuracy Acc=99.93 and sensitivity Sn=100% in the recognition of cardiac disorders. At the class level, the MPA-RF achieved a sensitivity of 100% for class F on the EDB database and ≥99.75 on the other two databases, although the class F had the minimum number of samples of ≤0.08% from the total number of class samples.

#### 3.3.4. Evaluation of the MPA-kNN

For the MPA-kNN classifier, the *K* parameter (number of nearest neighbors) was optimized. The MPA-kNN provided the lowest classification performance compared to the MPA-SVM, MPA-GBDT, and MPA-RF due to the characterization of the kNN as a lazy classifier depending on the distance for the classification process. However, the optimized version, MPA-kNN, performed well compared to the kNN itself. The detection accuracy of the AMMI classes with the MPA-kNN classifier was 94.96%, 95.40% and 92.14% on the MIT-BIH, EDB, and INCART database, respectively. As depicted in Table 7, it is clear that the MPA-kNN achieved an average Acc of 94:96%. Approximately ≤6.97% of the S class and ≤9.07% of the VEBs class were not classified correctly. Thus, according to the experimental results, we can conclude that the MPA-kNN performed well in terms of the classification accuracy.

**Figure 2:**
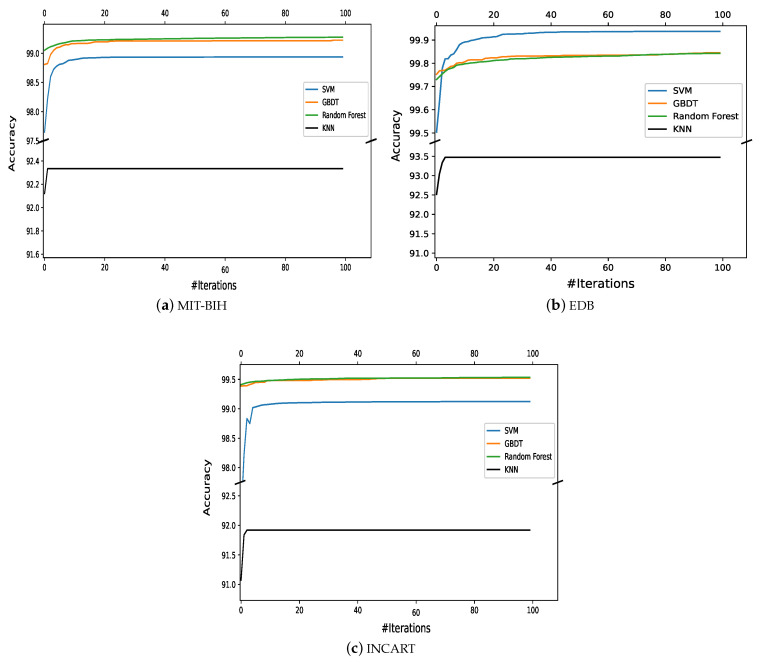
The convergence curves of the optimized classifiers on the three databases.

For more investigation, the convergence curves are presented for each optimized classifier on the MIT-BIH, EDB, and INCART datasets in Figure 2. The MPA-SVM higighted a high-speed convergence on the MIT-BIH database compared to the other models, while the MPA-kNN was in last place. On other two databases, the MPA-GBDT and MPA-RF had the highest speed convergence, and the MPA-kNN still had the least convergence. Moreover, the MPA-GBDT and MPA-RF had close convergence on the three databases.

In order to higight the improvement in the performance of the classifiers after optimization using the MPA algorithm, Table 8, Table 9, Table 10 and Table 11 show the improvements in the performance of each classifier with optimization (i.e., the classifier and its optimized version). The reported performance criteria were the average values of the Acc,Sn,Sp,Pr, and F1 on the three databases. The highest improvement was in the performance metrics of the SVM. Moreover, the improvement on the INCART database was higher than on the other two databases. Thus, it is clear that utilizing the proposed optimization algorithm improved the performance of these four ML classifiers significantly.

### 3.4. Comparison with Other Methods

The classification performance of the four optimized ML classifiers was compared to 16 of the state-of-the art methods, and the obtained results are reported in Table 12. In contrast, the results achieved by the previous other works were obtained for only five classes, of which four were known classes, and only one was unknown. The current proposed approaches accomplished average accuracies of 99.67%, 99.91%, 99.92%, and 97.07% on the EDB dataset for the MPA-SVM, MPA-GBDT, MPA-RF, and MPA-kNN, respectively. The MPA-SVM, MPA-GBDT, and MPA-RF achieved the highest percentages in terms of the ACC and Sn. Even the MPA-kNN, which was based on the lazy classifier kNN, performed well against the SVM, CNN, and kNN models in [44,45,46]. It can be concluded from Table 12 that the proposed method yielded a significantly improved classification performance in terms of the overall measurement factors compared to the other methods, which confirms the effectiveness of the proposed optimized classifiers.

## 4. Conclusions

This paper proposed an automatic arrhythmia classification method based on a new AI metaheuristic optimization algorithm and four ML classifiers for IoT-assisted smart healthcare systems. Multiclassifier models including the MPA-SVM, MPA-GBDT, MPA-RF, and MPA-kNN were introduced for classification with parameter optimization. The average classification accuracies achieved by the MPA with the SVM classifier were 99.48% (MIT-BIH), 99.90% (EDB), and 99.47% (INCART). The accuracies achieved by the MPA with the GBDT were 99.61% (MIT-BIH), 99.91% (EDB), and 99.72% (INCART); meanwhile, the MPA with the RF achieved 99.67% (MIT-BIH), 99.92% (EDB), and 99.73% (INCART), while the MPA with the kNN achieved 96.44% (MIT-BIH), 97.07% (EDB), and 93.51% (INCART). It is clear that the RF showed the most accurate results of these methods. Hence, it can be concluded that incorporating the MPA scheme can effectively optimize the ML classifiers, even a lazy one, such as the kNN. The achieved performance by the optimization step was ranked among the highest reported to date.

In future works, to enhance the ability to predict heart problems, other optimization algorithms can be investigated. For efficient methods to extract features and perform classification, it is necessary to incorporate the real-time surveillance of cardiac patients. Using powerful classification models (e.g., deep learning) is the possible next step of this research. To have meaningful classification outcomes with greater accuracy, these powerful classification models can be combined with the MPA algorithm, as it performed very well and enhanced the accuracy of the classification process. 

## Figures and Tables

**Table 1:** Total number of features extracted from the MIT-BIH, EDB, and INCART datasets.

Datasets	LBP	HOS + CM	HBF	DWT	RR	Total Number of Features
MIT-BIH	60	12 + 1	16	32	10	131
EDB	60	12 + 1	16	26	10	125
INCART	60	12 + 1	16	26	10	125

**Table 2:** The parameters of each classifier optimized using the MPA algorithm.

Classifiers	Parameters	Range
SVM	C	[0.0001,1000]
	Gamma	[0.0001, 1]
GBDT	Max_depth	[1, 13]
	Gamma	[0.0001, 1]
	Learning Rate	[0, 1]
RF	Max_depth	[1,13]
	Gamma	[0.0001, 1]
	Learning Rate	[0, 1]
kNN	K	[1,13]

**Table 3:** Details of the ECG databases used in the evaluation process.

Database	Subjects	Records	Leads	Location of Electrodes	Sample Rate	Resolution	Duration
MIT-BIH	48	48	12	Chest and limbs	360 HZ	11	30 min
EDB	79	90	2	Chest and limbs	250 HZ	12	120 min
INCART	32	75	12	Chest and limbs	257 HZ	12	30 min

**Table 4:** The performance of the MPA-SVM classifier on all the databases’ classes.

Database	MIT-BIH					EDB					INCART				
**Classes**	Acc	Se	Sp	Pr	F1	Acc	Se	Sp	Pr	F1	Acc	Se	Sp	Pr	F1
N	99.14	98.11	99.48	98.41	98.26	99.86	99.7	99.91	99.72	99.71	99.23	98.45	99.48	98.4	98.43
S	99.51	99.19	99.61	98.85	99.02	99.9	99.85	99.92	99.75	99.8	99.57	99.45	99.61	98.84	99.15
VEBs	99.51	98.4	99.89	99.67	99.03	99.18	97.93	99.61	98.83	98.38	99.9	99.58	100	100	99.8
F	99.78	99.9	99.74	99.21	99.55	99.93	99.97	99.92	99.75	99.86	99.92	99.9	99.93	99.77	99.84
Average	99.48	98.90	99.68	99.03	98.97	99.90	99.77	99.94	99.81	99.79	99.47	98.93	99.65	98.96	98.95

**Table 5:** The performance of the MPA-GBDT classifier on all the databases’ classes.

Database	MIT-BIH					EDB					INCART				
**Classes**	Acc	Se	Sp	Pr	F1	Acc	Se	Sp	Pr	F1	Acc	Se	Sp	Pr	F1
N	99.45	98.97	99.61	98.81	98.89	99.89	99.67	99.96	99.87	99.77	99.6	99.33	99.69	99.04	99.18
S	99.7	99.4	99.79	99.39	99.4	99.91	99.94	99.91	99.73	99.83	99.77	99.74	99.78	99.35	99.55
VEBs	99.62	98.49	100	100	99.24	**99.92**	99.49	**100**	**100**	**99.84**	99.58	98.59	**99.92**	**99.76**	99.17
F	99.69	99.73	99.68	99.04	99.39	99.9	**99.99**	99.88	99.63	99.81	**99.92**	**99.97**	99.91	99.71	**99.84**
Average	99.61	99.15	99.77	99.31	99.23	99.91	99.77	99.95	99.85	99.81	99.72	99.41	99.82	99.47	99.44

**Table 6:** The performance of the MPA-RF classifier on all the databases’ classes.

Database	MIT-BIH					EDB					INCART				
**Classes**	Acc	Se	Sp	Pr	F1	Acc	Se	Sp	Pr	F1	Acc	Se	Sp	Pr	F1
N	99.52	99.11	99.66	98.96	99.04	99.91	99.72	99.98	99.92	99.82	99.62	99.37	99.7	99.07	99.22
S	99.74	99.49	99.83	99.49	99.49	**99.93**	99.94	99.93	99.78	99.86	99.78	99.71	99.8	99.4	99.55
VEBs	99.66	98.73	**99.98**	**99.94**	99.3	**99.93**	99.6	**100**	**100**	**99.87**	99.61	98.75	99.91	99.73	99.24
F	**99.75**	**99.75**	**99.75**	99.24	**99.5**	99.92	**100**	99.9	99.69	99.84	**99.93**	**99.95**	**99.92**	**99.77**	**99.86**
Average	99.67	99.27	99.8	99.41	99.34	99.92	99.81	99.96	99.88	99.85	99.73	99.45	99.83	99.49	99.47

**Table 7:** The performance of the MPA-kNN classifier on all the databases’ classes.

Database	MIT-BIH					EDB					INCART				
**Classes**	Acc	Se	Sp	Pr	F1	Acc	Se	Sp	Pr	F1	Acc	Se	Sp	Pr	F1
N	94.03	80.78	98.37	94.18	86.97	94.73	81.29	**99.13**	**96.83**	88.38	89.82	73.63	95.11	83.11	78.09
S	96.36	97.57	95.96	89.04	93.11	96.66	99.19	95.8	88.95	93.79	93.03	98.58	91.17	78.95	87.68
VEBs	96.99	92.14	**98.65**	**95.9**	93.98	97.85	95.46	98.65	95.96	95.71	91.93	75.4	97.56	91.31	82.59
F	**98.39**	**99.72**	97.95	94.13	**96.85**	**99.02**	**99.9**	98.73	96.3	**98.07**	**99.24**	**99.85**	**99.04**	**97.19**	**98.5**
Average	96.44	92.55	97.73	93.31	92.73	97.07	93.96	98.08	94.51	93.99	93.51	86.87	95.72	87.64	86.72

**Table 8:** The performance comparison of the SVM and its optimized version, the MPA-SVM.

Database	MIT-BIH			EDB			INCART		
**Average**	**SVM**	**MPA-SVM**	**Improvement %**	**SVM**	**MPA-SVM**	**Improvement %**	**SVM**	**MPA-SVM**	**Improvement %**
Acc	91.79	99.48	7.69	91.31	99.90	8.59	87.36	99.47	12.11
Sn	83.73	98.90	15.17	82.63	99.77	17.14	75.0	98.93	23.93
Sp	94.55	99.68	5.13	94.23	99.94	5.71	91.61	99.65	8.04
Pr	89.41	99.03	9.62	89.65	99.81	10.16	87.21	98.96	11.75
F1	84.20	98.97	14.77	82.18	99.79	17.61	72.62	98.95	26.33

**Table 9:** The performance comparison of the GBDT and its optimized version, the MPA-GBDT.

Database	MIT-BIH			EDB			INCART		
**Average**	**GBDT**	**MPA-GBDT**	**Improvement %**	**GBDT**	**MPA-GBDT**	**Improvement %**	**GBDT**	**MPA-GBDT**	**Improvement %**
Acc	96.93	99.61	2.68	97.65	99.91	2.26	97.44	99.72	2.28
Sn	93.38	99.15	4.79	94.80	99.77	4.97	95.13	99.41	4.28
Sp	98.12	99.77	1.65	98.60	99.95	1.35	98.38	99.82	1.44
Pr	94.36	99.31	4.95	95.82	99.85	4.03	94.6	99.47	4.87
F1	93.84	99.23	5.39	95.29	99.81	4.52	94.88	99.44	4.56

**Table 10:** The performance comparison of the RF and its optimized version, the MPA-RF.

Database	MIT-BIH			EDB			INCART		
**Average**	**RF**	**MPA-RF**	**Improvement %**	**RF**	**MPA-RF**	**Improvement %**	**RF**	**MPA-RF**	**Improvement %**
Acc	96.90	99.67	2.77	97.65	99.92	2.27	97.5	99.73	2.23
Sn	93.37	99.27	5.9	94.83	99.81	4.98	94.75	99.45	4.7
Sp	98.09	99.8	1.71	98.86	99.96	1.1	98.42	99.83	1.41
Pr	94.26	99.41	5.15	98.61	99.88	1.27	95.27	99.49	4.22
F1	93.79	99.34	5.55	95.3	99.85	4.55	94.99	99.47	4.48

**Table 11:** The performance comparison of the kNN and its optimized version, the MPA-kNN.

Database	MIT-BIH			EDB			INCART		
**Average**	**kNN**	**MPA-kNN**	**Improvement %**	**kNN**	**MPA-kNN**	**Improvement %**	**kNN**	**MPA-kNN**	**Improvement %**
Acc	94.96	96.44	1.48	95.40	97.07	1.67	92.14	93.51	1.37
Sn	89.17	92.55	3.38	90.35	93.96	3.61	83.83	86.87	3.04
Sp	96.9	97.73	0.8	97.06	98.08	1.02	94.94	95.72	0.78
Pr	91.3	93.31	2.01	92.16	94.51	2.35	86.88	87.64	0.76
F1	89.71	92.73	7.14	90.56	93.99	3.43	83.83	86.72	2.89

**Table 12:** Classification performance comparisons between the proposed method and other methods.

Methods	#Classes	#Beats	Classifier	Acc %	Sn %
Roshan et al. [44]	5	34,989	SVM	93.50	**99.30**
Li et al. [47]	5	1800	SVM	97.30	97.40
Taiyong and Min [48]	4	100,688	RF	94.60	**98.51**
Serkan et al. [49]	5	83,648	CNN	**99.10**	93.91
Acharya et al. [45]	5	109,449	CNN	94.03	96.71
Yang et al. [50]	15	104986	KNN	97.70	-
Rishi et al. [46]	4	109,449	KNN	98.00	85.33
Shu-Lih et al. [51]	5	16,499	LSTM-CNN	98.10	97.50
Li et al. [52]	4	94,013	ResNet	**99.06**	93.21
Yildirim et al. [53]	13	833	1D-CNN	95.20	93.52
Oh et al. [54]	5	94,667	Modified U-Net	97.32	94.44
Marinho et al. [55]	5	100467	Bayes	94.30	-
Yang et al. [56]	15	3350	DL-CCANet	98.31	90.89
Plawiak et al. [57]	17	774	FGE	95.00	94.62
Patro et al. [58]	5	3551	PSO-GA-SVM	95.30	94.00
Qihang et al. [14]	8	6877	ATI-CNN	81.20	80.10
The proposed method	4	80,000	MPA-SVM	**99.48**	**98.90**
	4	80,000	MPA-GBDT	**99.61**	**99.15**
	4	80,000	MPA-RF	**99.67**	**99.27**
	4	80,000	MPA-kNN	96.42	92.55

## Data Availability

All used datasets are available free for public at https://www.kaggle.com/datasets.
